# Defining the metabolic signatures associated with human macrophage polarisation

**DOI:** 10.1042/BST20220504

**Published:** 2023-07-14

**Authors:** Adrián Povo-Retana, Rodrigo Landauro-Vera, Marco Fariñas, Sergio Sánchez-García, Carlota Alvarez-Lucena, Silvia Marin, Marta Cascante, Lisardo Boscá

**Affiliations:** 1 Instituto de Investigaciones Biomédicas Alberto Sols (CSIC-UAM), Arturo Duperier 4, 28029 Madrid, Spain; 2 Department of Biochemistry and Molecular Biomedicine-Institute of Biomedicine (IBUB), Faculty of Biology, Universitat de Barcelona, 08028 Barcelona, Spain; 3 CIBER of Hepatic and Digestive Diseases (CIBEREHD), Institute of Health Carlos III (ISCIII), 28029 Madrid, Spain; 4 Centro de Investigación Biomédica en Red de Enfermedades Cardiovasculares (CIBERCV), Institute of Health Carlos III (ISCIII), 28029 Madrid, Spain

**Keywords:** cell homeostasis, fibrosis, immunometabolism, inflammation, macrophages, monocytes

## Abstract

Macrophages are essential components of the innate immune system that play both homeostatic roles in healthy organs, and host defence functions against pathogens after tissue injury. To accomplish their physiological role, macrophages display different profiles of gene expression, immune function, and metabolic phenotypes that allow these cells to participate in different steps of the inflammatory reaction, from the initiation to the resolution phase. In addition, significant differences exist in the phenotype of macrophages depending on the tissue in which they are present and on the mammalian species. From a metabolic point of view, macrophages are essentially glycolytic cells; however, their metabolic fluxes are dependent on the functional polarisation of these cells. This metabolic and cellular plasticity offers the possibility to interfere with the activity of macrophages to avoid harmful effects due to persistent activation or the release of molecules that delay tissue recovery after injury.

## The innate immune system: macrophage (Mφ) activation and inflammation

The essence of the innate immune system has been tightly conserved through evolution. It includes, in addition to physical barriers such as the mucosa and skin, a series of myeloid cell subsets. Among them, Mφ and neutrophils are principal actors in inflammation. The origin of inflammation is diverse; it can be caused by endogenous factors (tissue repair and renewal, necrosis, bone rupture, etc.) or by exogenous challenges (mechanical, physical, chemical, immune insults or biological agents), and it is characterised by the accumulation of fluids and blood proteins (oedema) within the extracellular space and leukocytes that infiltrate in the lesion and become activated [1].

Inflammation can be divided into two different categories: acute or chronic inflammation, depending on the timespan of the response, the intensity, and the self-limitation of the process [1,2]. In acute inflammation, there is local vasodilation caused by the release of histamine and several bioactive molecules by mastocytes and other immune and non-immune cells that sense the injury. Due to the release of chemoattractant molecules (pathogen or damage-associated molecular patterns, PAMPs or DAMPs, respectively; cytokines and chemokines), neutrophils and monocytes are the first cell types that are recruited and infiltrate the lesion in a process called diapedesis (rolling, paving and docking that is mediated by ICAM-1, ICAM-2, E- and P-selectins) [1,3,4]. Monocytes are attached to the site of inflammation and they differentiate into Mφ to produce a wide range of biological mediators that orchestrate the inflammatory process; in the acute phase, pro-inflammatory antimicrobial mediators are released by Mφ and neutrophils (i.e. IFN-γ, IL-1β, IL-6 and reactive oxygen species among others), and phagocytosis is activated. Once the pathogen is eliminated or neutralised, the acute phase is followed by the resolution phase where the recruited Mφ secrete IL-10, VEGF, resolvins, lipoxins and other anti-inflammatory and pro-resolving lipid mediators that prevent chronic inflammation and promote the wound-healing process and the restoration of the normal cellular architecture of the tissue [5–7].

## Macrophages: diversity in origin and function

Macrophages, as innate immune cells, display multiple and non-redundant biological functions, from tissue homeostasis to host defence, antigen presentation and efferocytosis/phagocytosis of exogenous particles and dead cells [8]. Mφ have two main origins: they derive either from hematopoietic stem cells (HSC) of the embryonic yolk-sac, a primitive embryonic structure that will eventually perish and be replaced by the placenta, and which gives rise to tissue-resident Mφ [9,10], or from hematopoietic stem cells of the bone marrow. Human tissue-resident Mφ constitute numerous and diverse populations that exhibit self-renewal at low rates, which are long-lived cells that express specific cell-surface markers and patrol and scan the niche tissue where they are formerly nestled. They receive specific nomenclatures due to their particular transcriptomic, epigenetic signatures, and morphological aspects, which reflect the strong contribution of the environment to their functions; for example, splenic macrophages constitutively express genes involved in iron homeostasis. Macrophages receive specific nomenclatures depending on where they are located; (i.e. Kupffer cells in the liver, alveolar Mφ in the lungs, mesangial cells in the kidneys, microglia in the central nervous system, Langerhans cells in the epidermis, etc. [9–11]). Nevertheless, the repositioning of bone marrow-derived monocytes/Mφ into tissues that lost the resident population, allows them to regain the functions of the embryonic-derived Mφ. Furthermore, these newly allocated Mφ exhibit self-renewal capacities and exert the homeostatic functions typical of tissue-macrophages [12].

Additionally, in cases of acute inflammation and monocyte-overdemand, monocytopoiesis of extramedullary hematopoietic organs occurs to ensure appropriate Mφ availability to injured tissues (extramedullary haematopoiesis, EMH; i.e. spleen, liver, lymph nodes, etc.) [13]. Indeed, the identification of the pathways that are involved in EMH and the metabolic and functional roles of these cells deserve further study in the specific pathological conditions in which it occurs.

The monocytes derived from bone marrow precursors enter the blood via CC chemokine receptor 2 motifs (CCR2), arriving at different tissues where they differentiate into Mφ. Although murine and human monocytes share some common features, many differences have been established related to their function and phenotype; therefore, comparisons between them should be cautiously considered. Human monocytes are classified according to their CD14/CD16 expression level, and two main subsets are present in healthy individuals: the CD14^high^/CD16^low^ that is predominant, commonly referred to as classical macrophages, and a minor presence (<15%) of CD14^low^/CD16^+^, the non-classical Mφ and a lesser extent of CD14^high^/CD16^+^ cells [14]. These populations express specific patterns of adhesion molecules and chemokine receptors; for example, CD14^high^/CD16^low^ monocytes express CCR2, CX3CR1, CD62L and CD64, and they are found to be significantly increased in an inflammatory context. This diversity in the circulating monocytes precludes specific actions after infiltration of these cells at sites of inflammation. The terminal differentiation of monocytes into Mφ is mediated through the sensing of specific PAMPs signals such as toll-like receptors (TLRs) [15–18]. In addition, these cells are distributed and located in virtually all organs and tissues and, therefore, have a fundamental role in tissue homeostasis and regeneration in response to the presence of DAMPs that are released when the cell viability or function is compromised [19]. Indeed, Mφ are considered a bridge between innate and adaptive immunity when they act as antigen-presenting cells [20].

Therefore, Mφ are a heterogeneous population of immune cells that adapt and respond rapidly to inflammatory signals. In general, and simplistic terms, two main distinct subpopulations are defined among activated Mφ: classically activated Mφ towards a pro-inflammatory phenotype and, alternatively activated or anti-inflammatory Mφ [8,21–25]. The polarisation of Mφ is accompanied by drastic transcriptomic and metabolic reconfigurations [8]. Indeed, there is a current trend suggesting that re-education of Mφ into a specific phenotype could be crucial to finding new therapeutic strategies in the regulation of inflammation, from neurodegeneration to cancer. However, this goal requires more extensive and deeper knowledge of the molecular basis of Mφ polarisation [26].

## Macrophage activation and profiling

The acquisition of either a pro-inflammatory or anti-inflammatory phenotype by Mφ ultimately depends on the microenvironmental conditions at the site of inflammation. Therefore, when a pathogen or stressed cell is to be cleared, Mφ are activated to a pro-inflammatory profile [8,22]. Several specific molecules have been associated with inflammatory Mφ, such as TLR agonists, TNF-α, IL-1β and IFN-γ [8,22,27,28]. Once this function is fulfilled, the inflammatory Mφ, whose average viability in the tissue fluctuates between two or three days, are mainly cleared by the activation of apoptotic pathways, while cytokines are released (i.e. IL-10), which gradually facilitate the switch towards an anti-inflammatory and/or pro-resolving phenotype of the newly recruited naïve Mφ [24,29].

Importantly, inflammatory Mφ release reactive oxygen species (ROS), particularly superoxide (O_2_^-^) via the NADPH oxidases (mainly NOX2, and to a lesser extent NOX4) as part of the mechanism of host-defence [30–33]. In addition, these cells in rodents express NOS2 at the same time, which promotes a high synthesis of nitric oxide (NO) [34], whereas human macrophages (hMφ) have lost their ability to express NOS2 under these conditions. The reaction between NO and O_2_^-^ renders the synthesis of peroxynitrite, a potent oxidant molecule [35]. Because NO inhibits cytochrome oxidase and, therefore, the oxidative phosphorylation pathway, this constitutes a different immunometabolic regulation condition between humans and other species regarding Mφ biology [36,37]. Thus, from a metabolic and functional point of view, the main difference between inflammatory hMφ and other mammalian Mφ is the absence of high-throughput NO synthesis, which differentially influences the mitochondrial and energetic metabolism in these cells, depending on the species’ origin.

Regarding the alternative activation of Mφ, IL-4 and IL-13 are common inducers of anti-inflammatory Mφ polarisation [8,22,27,28]. These molecules are responsible for triggering the signalling cascades and transcription factor activation that induce the expression of pro-inflammatory or anti-inflammatory effectors. The profile of pro-inflammatory Mφ is mainly dependent on the activity of nuclear factor κB (NF-κB), interferon regulatory factor (IRF) 3, IRF5, signal transducer and activation of transcription (STAT) 1, STAT5, and activator protein 1 (AP-1) transcription factors, whereas anti-inflammatory Mφ turn on cascades that activate STAT6, IRF4, peroxisome proliferator-activated receptor-γ (PPARγ), liver X receptor (LXR), and Jumonji domain-containing 3 (JMJD3) transcription factors [8,22,38].

## Macrophage activation and immunometabolism

Mφ polarisation leads to metabolic reprogramming, where Mφ acquire polarisation-specific metabolic signatures [23,39]. Specifically, pro-inflammatory Mφ depend on glycolysis and the pentose phosphate pathway (PPP) to meet their energetic demands [24,40,41]. Glucose is metabolized into pyruvate, which is preferentially converted into lactate, even in aerobic conditions [24,42].

This elevated aerobic glycolysis provides precursors for *de novo* ribonucleotide and fatty acids biosynthesis, and aminoacid replenishment for protein synthesis. In addition, glycolysis ensures the rapid ATP production to meet the high energy requirements of phagocytosis, induces ROS production, and promotes the biosynthesis of specific cytokines [42]. Indeed, oxidative phosphorylation is impaired in pro-inflammatory Mφ, in part due to the synthesis of NO by NOS2 in those organisms that express this enzyme [34,43]. This metabolic rewiring is associated with additional HIF-1α activation, which enhances the glycolytic phenotype of pro-inflammatory Mφ through transcriptional mechanisms and constitutes a crucial node that integrates and modulates the metabolic demands, cell signalling, and immune function [39,44].

### Regulation of the fructose-6-phosphate/fructose-1,6-bisphosphate flow

**Figure 1.**
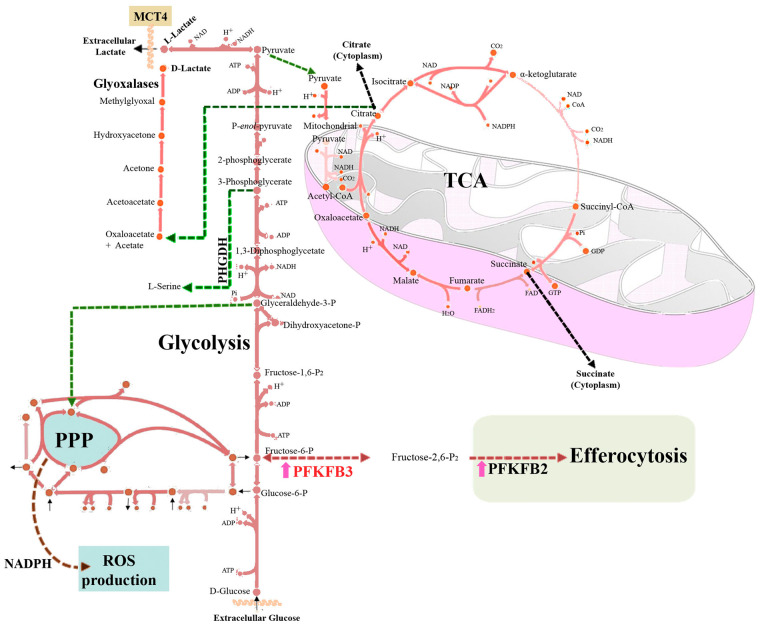
Pathways of the glycolytic and Krebs cycle in the functional polarisation of macrophages. Macrophages are essentially glycolytic cells. Under pro-inflammatory conditions, glucose consumption is increased primarily through enhanced hexose flux in which PFKFB3 is highly expressed. This promotes increased flux through 6-phosphofructo-1-kinase (PFK). Furthermore, PFKFB2 expression levels contribute to and are essential for efferocytosis/phagocytosis of macrophages, suggesting a compartmentalisation of these isoenzymes. Due to this high hexose flux, some alternative diversions can occur at the triose-phosphate level, either towards phosphoglycerate dehydrogenase (PHGDH, from 3-phosphoglycerate) expression in anti-inflammatory macrophages, resulting in increased serine biosynthesis, or from the pro-inflammatory pathway that involves the synthesis of methylglyoxal and the accumulation of the detoxified end-product d-lactate. The Krebs cycle in macrophages has at least two main outflows at the level of citrate and succinate. ROS, reactive oxygen species; PPP, pentose phosphate pathway; MCT4, monocarboxylate transporter 4.

Up-regulation of the glycolytic flux in Mφ is very dependent on the expression of the 6-phosphofructo-2-kinase/fructose-2,6-bisphosphatase isoenzymes (PFKFB1–4) [22,43]. These isoenzymes maintain tight control on the futile cycle involved in the synthesis and degradation of Fru-2,6-P_2_, a potent activator of 6-phosphofructo-1-kinase (PFK), the enzyme that catalyses the phosphorylation of Fru-6-P to Fru-1,6-P_2_ in the glycolytic pathway. The activity of PFK is inhibited by acidic pH, ATP and citrate. However, Fru-2,6-P_2_ provokes a conformational change that impairs this inhibition. Therefore, the precise control of Fru-2,6-P_2_ concentration appears to be a key regulator of the glycolytic flux in Mφ. Regarding PFKFB isoenzymes, they are bifunctional enzymes consisting of the fusion of two separated domains, one containing the kinase activity and another carrying out the bisphosphatase activity, but their specific activities vary across the isoenzymes that also exhibit cell-dependent expression pattern. In resting and tissue Mφ the main expressed isoenzyme is PFKFB1, characterised by a dominant bisphosphatase *vs*. kinase activity, thus maintaining low levels of the effector Fru-2,6-P_2_, which contributes to the low glycolytic rate observed in these resting cells [7]. However, upon pro-inflammatory activation, Mφ express high levels of the PFKFB3 isoform due to its dependence on HIF-1α transcriptional activity. This isoform has a dominant kinase *vs*. bisphosphatase activity, which results in a rise of Fru-2,6-P_2_ and enhances the glycolytic flux. Silencing PFKFB3 or selective inhibitors of this enzyme drastically reduce the glycolytic flux of pro-inflammatory Mφ, leading to a rapid loss in cell viability [43,45]. Interestingly, a pivotal role for PFKFB2 expression and lactate accumulation in efferocytic Mφ has recently been described. This is an alternative detour of glycolysis carried out mainly by anti-inflammatory Mφ in order to act as professional phagocytic cells, promote the correct clearance of apoptotic cells, and facilitate tissue regeneration [46]. Together, these data reveal the relevant role of the PFKFB isoenzymes in the biological function of Mφ (Figure 1).

### TCA cycle and oxidative phosphorylation in Mφ

Pro-inflammatory Mφ also exhibit an altered Krebs cycle, which is disrupted at several steps, leading to citrate and succinate export into the cytoplasm, where these metabolites play several regulatory roles [47]. The citrate and succinate outflows in the Krebs cycle are mainly due to the inhibition of succinate dehydrogenase by itaconate, which in turn promotes the inverted electron transport towards OXPHOS complex I and facilitates mitochondrial ROS production as well as an increase in succinate that favours additional pro-inflammatory signalling. In the cytoplasm, succinate induces the activation of HIF-1α, which contributes to maintaining the pro-inflammatory response [22,48]. In addition, it has been observed that cytoplasmic export of succinate by pro-inflammatory Mφ leads to protein succinylation (i.e. glyceraldehyde-3-phosphate dehydrogenase and malate dehydrogenase), although the effects of this posttranslational modification are yet unknown [49]. In fact, several Krebs cycle intermediates escape the mitochondria and exert regulatory actions (itaconate, citrate, succinate; Figure 1) [23,39].

Concerning TCA metabolic flows, citrate can also be converted into oxaloacetate and acetyl-CoA via the ATP-citrate lyase [50], being used as a source for histone acetylation, thus leading to epigenetic changes that contribute to the polarisation process [51]. In general, not only does acetyl-CoA regulate histone acetylation but it also regulates post-transcriptional protein modifications that alter hMφ polarisation. For example, by modifying Cys-30 acetylation of the RelA/p65 subunit of NF-κB, the presence of acetyl-CoA favours the pro-inflammatory hMφ activity [52]. Moreover, citrate can be redirected into the Krebs cycle as malate by malate dehydrogenase.

Regarding the bioenergetics of anti-inflammatory Mφ, they are more dependent on oxidative phosphorylation, using glucose as an electron supplier through canonical glycolysis and the Krebs cycle [53]. Moreover, α-ketoglutarate is a repressor of inflammation by inhibiting NF-κB transcriptional activity [54]. Thus, the succinate/α-ketoglutarate ratio is used to study the balance of pro-/anti-inflammatory Mφ polarisation (Figure 1).

### Metabolic detours in Mφ

An increase in the glycolytic flux is a usual metabolic condition that favours flux detours towards glycolysis-accessory pathways. In Mφ, two of these metabolic cascades are the 3-phosphoglycerate dehydrogenase (PHGDH) and the methylglyoxal pathways, which impact Mφ polarisation [55,56]. The PHGDH pathway is at the crossroad of Mφ polarisation: the activity is repressed under pro-inflammatory conditions and is up-regulated in response to IL-4 treatment of Mφ (anti-inflammatory polarisation) [55]. In addition, PHGDH allows *de novo* synthesis of serine from the glycolytic intermediate 3-phosphoglycerate. Particularly, serine appears to contribute to the expression of several pro-inflammatory cytokines like IL-1β, through the one-carbon metabolism [57].

Another pathway derived from enhanced glycolysis is the methylglyoxal/D-lactate detour. Methylglyoxal is a highly reactive compound that comes from dihydroxyacetone-phosphate and glyceraldehyde-3-phosphate glycolytic intermediates. It has been postulated that pro-inflammatory Mφ produce methylglyoxal, thus leading to sustained inflammation and cell stress through paracrine effects [55,58]. Methylglyoxal production results in the glycation of reducing sugars and amino residues present in several macromolecules, like proteins, lipids, and nucleic acids. This glycation process leads to the generation of advanced glycation end-products (AGE), which induces oxidative stress and even apoptosis [58]. However, methylglyoxal is metabolised by the glyoxalase system which is also under transcriptional control [59]. This system consists of two sequential reactions in which methylglyoxal is converted to S-d-lactoyl-glutathione and finally into d-lactate, being the first protective reaction against AGE formation (Figure 1).

## Concluding remarks

The plasticity of the monocyte/Mφ cell lineage and the specific metabolic dependence of the polarisation phenotypes offer the possibility to induce or modulate a metabolic rewiring either by using activators or inhibitors of checkpoint enzymes that divert the metabolic fluxes associated to the polarisation phenotype.

## Perspectives

- Macrophages are important players in innate immunity carrying out multiple functions, from host defence against pathogens to antigen presentation, tissue homeostasis, and bone dynamics that can be modulated by interfering their metabolism.
- The diversity in macrophage functions varies from tissues and mammalian species, which determines their different functional roles and the adaptations occurring across evolution.
- Understanding the metabolic regulation of macrophages under the different functional polarisations of these cells might open novel strategies to modulate their activity and to avoid harmful effects due to dysregulation of their fate, such as occurs under chronic inflammation.

## Abbreviations

AGE: advanced glycation end-products
CCR2: CC chemokine receptor 2 motifs
EMH: extramedullary haematopoiesis
NO: nitric oxide
PFK: 6-phosphofructo-1-kinase
PHGDH: 3-phosphoglycerate dehydrogenase
PPP: pentose phosphate pathway
ROS: reactive oxygen species
